# Psychometric evaluation of the Perceived Parental Phubbing Scale (PPPS) among Iranian university students: associations with psychosocial factors and group differences

**DOI:** 10.1186/s12889-026-26498-y

**Published:** 2026-02-04

**Authors:** Mehran Mohammadi, Nikzad Ghanbari

**Affiliations:** Department of Psychology, Faculty of Psychology and Educational Sciences, Shahid Beheshti University, Tehran, Iran

**Keywords:** Parental phubbing, Smartphone addiction, Fear of missing out (FoMO), Loneliness, Psychometric validation, Iranian university students

## Abstract

**Background:**

Parental phubbing, the experience of children perceiving parental inattentiveness due to smartphone use, can signal emotional unavailability, perceived rejection, and model maladaptive behaviors. Theoretically, Risky Families Model highlights how emotionally unavailable parenting disrupts children’s emotional and social development, PAR theory explains children’s psychological responses to perceived parental neglect, and Social Learning Theory describes how children may adopt parents’ smartphone habits. Despite these theoretical foundations, most research has been conducted in Chinese populations, limiting cross-cultural generalizability, and no validated Persian-language instrument exists. This study aimed to translate, culturally adapt, and validate the Persian Perceived Parental Phubbing Scale (PPPS) among Iranian university students, and to examine its psychosocial and sociodemographic correlates.

**Methods:**

A cross-sectional online survey was conducted with 428 Iranian university students (70.8% female; *M* = 28.03, *SD* = 8.67). The participants completed the adapted 9-item PPPS alongside validated measures of smartphone addiction (SABAS), loneliness (ULS-8), and fear of missing out (FoMOs). Confirmatory factor analysis (CFA) was used to evaluate structural validity, whereas Cronbach’s alpha (*α*), McDonald’s omega (*ω*), and Guttman’s lambda (*λ*) were used to assess reliability. Group differences were analysed via t tests and ANOVA.

**Results:**

Confirmatory factor analysis supported a unidimensional structure with acceptable model fit (RMSEA = 0.076, CFI = 0.98), and the PPPS showed high internal consistency (*α* = 0.90, *ω* = 0.901). The PPPS score was positively correlated with smartphone addiction (*r* = .29), loneliness (*r* = .27), and FoMO (*r* = .42). Higher scores were found among students with psychological disorders and those in romantic relationships. No significant differences emerged in terms of gender, residence, educational level, or family history of psychological disorders.

**Conclusions:**

The Persian PPPS is a valid and reliable tool for assessing perceived parental phubbing among Iranian students. The lack of gender differences suggests cultural or developmental nuances in how such behaviors are interpreted. The findings highlight the role of psychological vulnerability and relational context, offering a foundation for future cross-cultural research and culturally responsive interventions promoting mindful smartphone use in families.

**Supplementary Information:**

The online version contains supplementary material available at 10.1186/s12889-026-26498-y.

## Background

In recent years, digitalization has introduced new behavioral patterns in individuals’ social interactions. While mobile phones provide many conveniences, they can also undermine the quality of interpersonal interactions [1–4]. For example, increased mobile phone use is linked to a decline in the perceived quality of social connectedness [5]. Supporting this, an experimental study conducted with young adult participants found that participants who had access to their phones while interacting with unacquainted peers reported lower enjoyment and socialized significantly less compared to those without phone access, indicating that smartphones can diminish the well-being derived from social interactions [6].

This trend has raised concerns about its potential negative social consequences, particularly for young people, who are profoundly influenced by digital technologies [7]. According to the International Telecommunication Union [8], approximately 80% of the global population over the age of 10 owns a mobile phone. Consistent with this global pattern, a 2024 study conducted on a broad Iranian population aged 6–60 years reported that all participants were smartphone users, with an average daily usage of approximately six hours [9].

The widespread integration of smartphones into everyday life has increased the likelihood of attentional disengagement during interpersonal communication, a phenomenon commonly referred to as phubbing [1, 10–12]. Phubbing is a combination of the words’ phone’ and ‘sobbing’, and it refers to situations in which an individual is ignored during an interaction because their conversation partner prioritizes smartphone use [10, 13], which may lead the ignored person to feel excluded or devalued by the phubber [14–16].

### Parental phubbing as a new emerging issue in the context of family

Smartphone use during parenting has become increasingly common in recent years [17–19]. Research indicates that more than 90% of parents aged 18–49 years who have infants or toddlers are active smartphone users [3]. A study conducted by McDaniel et al. [20] on 264 parents revealed substantial variability in parental smartphone use. While low-use parents reported using their phones for approximately 2.4 hours per day, representing an estimated 13% of the time spent with their child, high-use parents reported up to 8 hours of daily phone use, accounting for nearly half of their caregiving time. These findings have contributed to growing concerns regarding distracted parenting [3], a term that may reflect a shifting paradigm in contemporary parenting practices.

Although phubbing has been widely examined in various interpersonal contexts [21, 22], most previous studies have focused on partner phubbing in romantic relationships [11, 23] Given the central role of the family in shaping children’s socioemotional development, examining phubbing in parent–child interactions is particularly important [24–26]. Disruptions in communication and parental responsiveness can have long-lasting consequences for childhood adjustment, family functioning, and even the quality of future romantic relationships [27–30].

One key contributor to these disruptions is parental phubbing, which is defined as the experience in which children perceive that their parents are neglecting face-to-face interactions during conversations by focusing on mobile phones [31]. Put differently, parental phubbing captures the child’s sense that the parent is so absorbed in their phone that they fail to provide adequate attention [32]. As parents represent a primary source of meaning, emotional security, and social support, parental phubbing may undermine children’s fundamental need to belong and is associated with lower levels of life satisfaction [2, 3, 18, 28, 33].

Research on parental smartphone use has introduced overlapping concepts, including parental phubbing, technoference, parental screen distraction, and co-present smartphone use [19]. While superficially similar, these terms reflect distinct aspects of parental phone engagement. Parental phubbing is a child-centered form of technoference that emphasizes the subjective experience of being ignored [19], whereas technoference refers more broadly to any technological interference that may impede parent–child interactions or family communication [34]. This differentiation draws attention to the importance of accurately specifying the conceptual focus of the construct.

### Theoretical and conceptual background

Parental smartphone use can adversely affect parental responsiveness by reducing parents’ sensitivity to children’s bids for attention and by weakening the quality of parent–child interactions, particularly when parents are deeply engrossed in their devices [35]. When such patterns of attentional disengagement occur repeatedly, children may interpret them not merely as situational distractions but as signals of emotional unavailability. Accordingly, parental phubbing has been conceptualized as a subtle form of relational neglect with potential implications for children’s emotional and behavioral functioning [36]. The Risky Families Model [37] provides a useful framework for understanding these effects. This model proposes that family environments characterized by emotional unavailability, conflict, or neglect can disrupt children’s stress-response systems, emotion regulation, and social competence. Within this perspective, perceived parental phubbing may function as a contemporary form of social exclusion and a family-level risk factor for psychological maladjustment and developmental needs [1]. Empirical findings support this interpretation, showing that perceived parental phubbing is associated with social withdrawal [1, 38], negative emotional states [1, 12, 28], sleep quality problems [12] and academic burnout [10, 39–41]. Research has also linked phubbing to externalizing problems, including parent–child conflict [1, 42], aggression [14, 38, 43] and bullying behaviors [28, 44, 45] among adolescents and young adults.

Beyond its family-level implications, parental acceptance–rejection theory (PAR theory) clarifies the psychological mechanisms through which parental phubbing may exert its influence on interpersonal relationships and suggests that individuals who perceive themselves as rejected are more vulnerable to psychological distress compared to those who experience acceptance [46]. Consistent with this model, parental phubbing may be understood as a form of neglect, which has been shown to predict parental indifference over time [47]. Given the symbolic nature of rejection within this theory and the well-documented perceptual discrepancies between parents and children regarding phubbing behaviors [48], parental phone distraction can be theoretically understood as a symbolic cue of rejection, even when such behavior is unintentional. Empirical findings support this interpretation, demonstrating associations between perceived parental phubbing and internalizing symptoms such as depression [2, 49–56], anxiety [10, 39, 45, 55, 57], low self-esteem [58–60], loneliness [25, 31, 53, 61, 62] as well as relational outcomes such as lower levels of reciprocal filial piety (RFP) behaviors as a consequence of parental inacceptance [63].

Complementing these family-based and perceptual accounts, children’s behavioral responses to parental phubbing can also be understood through learning-based processes. Social Learning Theory provides a behavioral framework for understanding children’s adaptation to parental phone use, suggesting that children internalize the observable behaviors of their parents (such as phubbing) as socially acceptable norms [32, 58, 64]. Accordingly, perceptions of high parental smartphone use can serve as a notable cue, prompting children to model these behaviors and potentially leading to their own maladaptive technology use, including internet addiction [58], problematic smartphone use [27, 40, 48, 59, 64, 65], problematic gaming [32, 47, 60], and emerging issues such as short-form video addiction [55]. In contrast, recent studies have argued that until parents demonstrate moderate use of technology, children are more inclined to mirror those same habits [32]. These findings underscore the role of observational learning in shaping children’s technology habits.

Taken together, these complementary theoretical perspectives underscore the multifaceted nature of parental phubbing and provide a solid foundation for understanding its mechanisms and potential consequences. By integrating the Risky Families Model, PAR theory, and Social Learning Theory, we gain deeper insights into how parental phubbing functions as a multi-level risk factor that impacts children’s emotional, perceptual, and behavioral outcomes, highlighting the need for valid and reliable measurement tools to accurately capture individuals’ subjective experiences of being phubbed.

### Current study

Although research increasingly links parental phubbing to various psychosocial and behavioral outcomes [30], critical gaps remain, constraining progress in the literature. In particular, Most empirical evidence originates from Chinese samples, raising concerns about the cross-cultural generalizability of the findings [12, 32, 44, 58, 62, 66–70]. Because parenting practices, technology-use patterns, and family dynamics vary across cultures [67, 71], investigations in other populations are necessary to determine whether parental phubbing exerts similar effects or whether contextual factors influence its impact.

Moreover, despite strong theoretical foundations, no validated instrument is currently available for assessing perceived parental phubbing in Middle Eastern cultural contexts. The availability of a reliable, culturally appropriate tool is essential for examining risk factors, identifying vulnerable groups, and supporting interventions aimed at improving family dynamics. Such a tool can guide psychoeducation, enhance parental self-monitoring, and ultimately promote children’s emotional and behavioral development within the family [3].

Additionally, while research on parental phubbing has grown, limited attention has been paid to sociodemographic factors that influence perceptions of phubbing, leaving uncertainty about which groups may be particularly vulnerable. To address this gap, the present study also explored sociodemographic differences, aiming to identify particularly susceptible groups and lay the groundwork for interventions tailored to these populations.

In response to these gaps, the present study aimed to translate and validate the first short scale for measuring perceived parental phubbing in a Middle Eastern context. The study specifically focused on Iranian university students, a population frequently exposed to parental smartphone use in everyday family dynamics. This makes them capable of reliably reporting their experiences of parental distraction [13]. This focus expands prior work by moving beyond adolescent samples and taking an initial step toward broader developmental measurement [30, 72].

To achieve this aim, we adopted the modified one-dimensional version of the Partner Phubbing Scale (Pphubbing) developed by Roberts and David [13], which has shown acceptable psychometric properties in measuring perceptions of being phubbed by a romantic partner. Consistent with prior research, we adapted the nine items by replacing the word “partner” with “parent” [67]. Ultimately, this study aims to address the following hypotheses:

- H1: The Persian version of the Perceived Parental Phubbing Scale (PPPS) will demonstrate acceptable psychometric properties, including construct validity, internal consistency, and satisfactory model fit indices.
- H2: Perceived Parental Phubbing Scale (PPPS) scores will significantly differ across individual characteristics, including gender, marital status, psychological disorder history, educational level, and place of residence.

## Method

### Participants

This cross-sectional study recruited 428 Iranian university students (303 females, 125 males; M = 28.03 years, SD = 8.67) through convenience sampling between May 14 and June 9, 2025. Participants were eligible to take part in the study if they met the following inclusion criteria: (1) being 18 years of age or older (2), being currently enrolled as a university student, and (3) willingness to participate voluntarily. The exclusion criteria were: (1) failure to provide informed consent and (2) submitting incomplete responses.

### Measures

In addition to answering a short demographic questionnaire, participants responded to the following set of instruments:

### Perceived Parental Phubbing Scale (PPPS)

The Perceived Parental Phubbing Scale (PPPS) was adapted from the 9-item Partner Phubbing Scale developed by Roberts and David [13], which assesses the extent to which an individual perceives their romantic partner as being distracted by their phone during interactions. The original scale demonstrated acceptable psychometric properties and was developed through rigorous item generation, pretesting, and both exploratory and confirmatory factor analyses. While Cronbach’s alpha was not explicitly reported in the original study, the construct reliability was estimated at 0.93, serving a similar purpose in confirming the internal consistency of the scale. In the present study, the internal consistency of the adapted Persian version of the scale was examined, yielding a Cronbach’s alpha of 0.90, which indicates good internal reliability. The wording of the items was modified to reflect a parental rather than a romantic relationship context. The participants rated the frequency of their parents’ phubbing behavior on a 5-point Likert scale ranging from 1 (never) to 5 (all the time). Notably, the original scale included one reverse-worded item (Item 7: “My partner does not use his or her phone when we are talking”), yet the authors did not provide a clear rationale for its inclusion. When adapting the scale, we chose not to retain reverse scoring. This decision was based on empirical evidence indicating that although reverse-worded items are traditionally used to control for acquiescence bias, they often introduce confusion, reduce response consistency, and may compromise internal validity, especially among general population samples [30, 73–75]. Instead, this item was reworded to ensure consistent response direction, thus improving clarity and engagement (see items in Additional file 1).

### Fear of Missing Out Scale (FoMOs)

Fear of missing out was assessed via the 10-item Fear of Missing Out Scale (FoMOs) developed by Przybylski et al. [76]. This self-report measure was constructed through a rigorous, data-driven process based on a large and diverse international sample, utilizing latent trait theory to select the most representative items. The respondents rated each item on a 5-point Likert-type scale ranging from 1 (Not at all true of me) to 5 (Extremely true of me), with instructions emphasizing honest responses on the basis of actual experiences rather than idealized experiences. Sample items include “I get worried when I find out my friends are having fun without me” and “It bothers me when I miss an opportunity to meet up with friends”. The scale has demonstrated good psychometric properties, including good internal consistency (*a* = 0.89). In Iran, the Persian version of the FoMOs has also been validated by Chashmi et al. [77] among university students, demonstrating good reliability (*a* = 0.91). Confirmatory factor analysis supported the one-factor structure of the scale in the Iranian student population.

### The UCLA loneliness scale short form (ULS-8)

Loneliness was assessed via the 8-item short form of the University of California, Los Angeles Loneliness Scale (ULS-8). The ULS-8 was developed by Hays and Dimatteo [78] as an abbreviated version of the original 20-item UCLA loneliness scale and was designed to maintain the psychometric integrity of the longer form while reducing administration time. This measure aims to assess feelings of loneliness and social isolation. Participants respond to each item via a 4-point Likert-type scale, with responses typically ranging from 1 (“Never”) to 4 (“Often”).

The scale includes both positively and negatively worded items to reduce response bias. Example items include “People are around me but not with me” and “I feel left out”. In its original validation, the scale showed good internal consistency (*a* = 0.84). The ULS-8 scores were well distributed, ranging from 0% to 100% of the maximum possible score, indicating good variability. The ULS-8 was highly correlated with the full 20-item ULS-20 scale (*r* = .91), suggesting that it is a valid short-form alternative. Overall, the results indicate that the ULS-8 is a reliable, valid, and practical short-form measure of loneliness.

### Smartphone Application-Based Addiction Scale (SABAS)

The risk of smartphone application-based addiction was assessed via the 6-item Smartphone Application-Based Addiction Scale (SABAS), which was first developed by Csibi et al. [79] and validated in English by Csibi et al. [80]. This self-report tool is designed for quick screening of SABAS symptoms. The participants rated each item on a 6-point Likert-type scale ranging from 1 (“Strongly Disagree”) to 6 (“Strongly Agree”). The total score ranges from 6 to 36, with higher scores indicating a higher risk of SABAS. Sample items include “My smartphone is the most important thing in my life” and “Over time, I fiddle around more and more with my smartphone”. The SABAS demonstrated good internal reliability, with a Cronbach’s alpha of 0.81. The scale also showed good convergent validity, with significant correlations with the Nomophobia Questionnaire (*r* = .63, *p* < .01) and the Deprivation Sensation Scale (*r* = -.60, *p* < .01). The SABAS validated and demonstrated a unidimensional structure in Iranian adolescents, and psychometric evaluation revealed good internal consistency (*a* = 0.86), composite reliability (0.88), and test-retest reliability (*r* = .83).

### Procedure

Data collection was carried out using an online questionnaire hosted on Porsline, a widely used Iranian survey platform comparable to Google Forms. The survey link was disseminated through Telegram and WhatsApp groups specifically created for university students (e.g., departmental groups, student discussion channels, and university-related academic groups). This recruitment method ensured that only individuals who were currently enrolled as university students had access to the questionnaire.

At the beginning of the survey, participants were informed that only currently enrolled university students were eligible to participate. This eligibility requirement was explicitly stated to prevent non-students from entering or completing the survey. Before proceeding, participants were provided with an overview of the study’s objectives, confidentiality assurances, and their rights as research participants. Informed consent was obtained electronically by asking participants to check a consent box. Completing the questionnaire took approximately 5–10 min.

The demographic section included questions on gender, age, marital status, place of residence, and educational status, as well as items regarding personal or immediate family history of psychological disorders (see Additional File 2 for the complete list of demographic items). The main section consisted of four standardized self-report instruments: the Perceived Parental Phubbing Scale (PPPS), the Fear of Missing Out Scale (FoMOs), the Smartphone Application-Based Addiction Scale (SABAS), and the UCLA Loneliness Scale – Short Form (ULS-8).

Prior to the main data collection, a pilot study was conducted to evaluate the clarity, linguistic accuracy, and cultural appropriateness of the Persian-translated PPPS. Using a standard forward–backward translation procedure, the preliminary version was administered to 10 individuals who met the inclusion criteria. Based on their feedback, minor revisions were made to improve clarity and contextual relevance. After finalizing the Persian version of the PPPS, the complete survey was administered to a larger sample of 428 university students.

### Data analyses

This research involved five distinct stages of data analysis. First, the preliminary statistical assumptions, particularly those required for Confirmatory Factor Analysis (CFA), were examined. These included multivariate normality, linearity, and homoscedasticity. The Kolmogorov-Smirnov test, along with Skewness and Kurtosis values, was used to assess normality. Results showed that all variables were normally distributed (*p* > .05), and the skewness and kurtosis values were within ± 2, indicating acceptable normality (see Table 1).

**Table 1:** Psychometric properties of the perceived Parental Phubbing Scale (PPPS): factor loadings and item analysis

Items statistics							Item-Total statistics			
Items		Mean	SD	F. L	V		I.T.	C.D.	Skewness	Kurtosis
Item1		1.67	0.93	0.58	39.91		0.57	0.90	1.31	0.95
Item2		1.83	1.05	0.57	38.64		0.59	0.90	1.20	0.73
Item3		1.80	0.98	0.75	37.51		0.75	0.88	0.99	0.03
Item4		2.29	1.17	0.59	37.91		0.56	0.90	0.57	-0.58
Item5		1.96	1.03	0.79	37.38		0.72	0.89	0.88	0.11
Item6		1.87	1.00	0.77	37.81		0.70	0.89	1.03	0.46
Item7		1.78	0.98	0.82	37.21		0.78	0.88	1.27	1.22
Item8		1.96	1.04	0.76	37.63		0.68	0.89	1.02	0.40
Item9		1.97	1.05	0.74	37.61		0.68	0.89	0.90	0.11
PPPS		17.13	6.88	-	-		-	-	0.81	-0.01

*V* Scale variance if the item is deleted, *F.L.* Factor loading, *I.T*. Corrected item–total correlations, *C.D*. Cronbach’s alpha if the item is deleted, *PPPS* Perceived Parental Phubbing Scale

Second, descriptive statistics were calculated for the Perceived Parental Phubbing Scale (PPPS), including means, standard deviations, item factor loadings, and score ranges.

Third, CFA was performed using the Maximum Likelihood (ML) method to assess the internal structure of the PPPS. Fit indices including RMSEA, SRMR, CFI, NFI, AGFI, RFI, IFI, and PNFI were used to evaluate model fit.

Fourth, internal consistency reliability of the PPPS was evaluated using data from the main sample. The reliability coefficients included Cronbach’s alpha, McDonald’s omega, and Guttman’s lambda.

Finally, Pearson correlation coefficients were calculated to examine the convergent validity of the PPPS with theoretically related constructs. Loneliness reflects emotional responses to interpersonal problems resulting from parental neglect or reduced interaction [62, 81]. FoMO captures cognitive–social vulnerabilities associated with the need for social connection [31], and smartphone addiction represents behavioral outcomes linked to parental phubbing [31, 82]. Collectively, these constructs cover emotional, cognitive–social, and behavioral domains, providing a comprehensive basis for testing convergent validity. All the statistical analyses were conducted using SPSS version 26 and LISREL version 8.0.

## Results

### Descriptive statistics

A total of 428 Iranian university students participated in the study, comprising 303 females (70.8%) and 125 males (29.2%), with a mean age of 28.03 years (SD = 8.67). In terms of marital status, 44.2% were single, 47.4% were married, 7.2% were in a relationship, and 1.2% were divorced. With respect to educational status, 26.9% held an associate degree, 53.0% held a bachelor’s degree, 18.9% held a master’s degree, and 1.2% held a PhD. Most participants (89.0%) resided in urban areas, whereas 11.0% lived in rural areas. Concerning mental health, 95.3% reported no history of psychological disorders, whereas 4.7% reported having one. Additionally, 93.9% of the participants reported no family history of psychological disorders, whereas 6.1% reported a positive family history.

### Factor structure

**Fig. 1:**
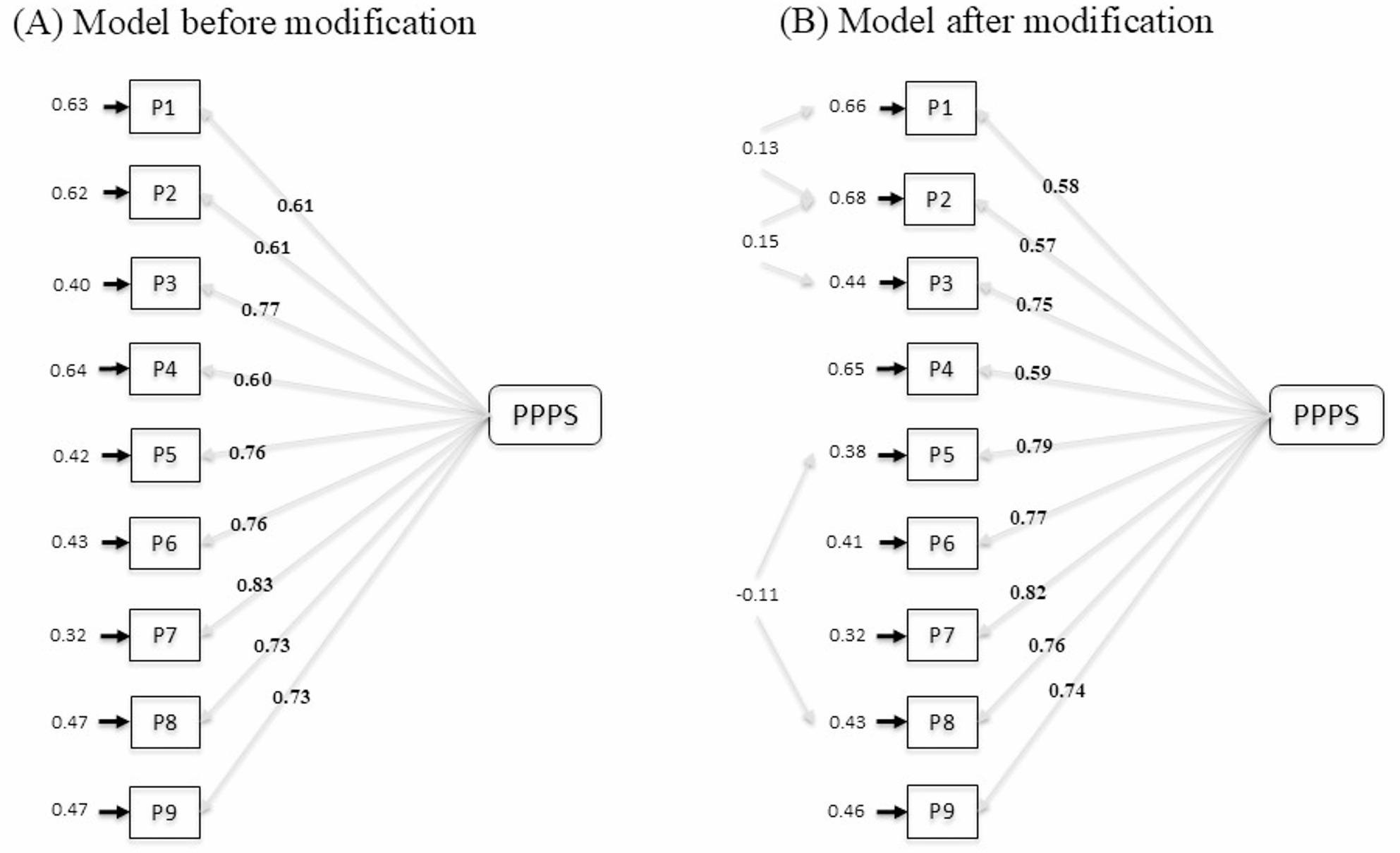
Standardized factor loadings of the Persian version of the Perceived Parental Phubbing Scale (PPPS). Panel **A** presents the model before modification (χ² = 150.73, df = 27, *p* < .001, RMSEA = 0.103), and Panel **B** presents the model after modification (χ² = 82.87, df = 24, *p* < .001, RMSEA = 0.076)

All factor loadings were statistically significant (*p* < .001) and exceeded the recommended threshold of 0.55, ranging from 0.57 to 0.82, indicating satisfactory item representation of the latent construct (see Table 1; Fig. 1). Descriptive statistics for the nine PPPS items revealed a mean score of 1.90 (*SD* = 1.024), with individual item means ranging from 1.67 to 2.29 (Table 1). The goodness-of-fit indices supported the single-factor structure of the PPPS. According to the recommended criteria [83], acceptable model fit is indicated by RMSEA < 0.08, CFI > 0.90, NFI > 0.90, and RMR < 0.08. In the present study, the fit indices were as follows: RMSEA = 0.076 (90% CI = 0.058–0.094), CFI = 0.98, NFI = 0.98, IFI = 0.98, RFI = 0.97, GFI = 0.96, AGFI = 0.87, and RMR = 0.036, all indicating good model fit (see Table 2).

**Table 2:** Confirmatory Factor Analysis (CFA) fit indices for Single-Factor model: before and after modification

Goodness-of-Fit index	Model Fit Single-factor BM	Model Fit Single-factor AM	Recommended Value	Decision
RMSEA (CI 90%)	0.103 (0.091–0.12)	0.076 (0.058–0.094)	*≤ 0.08*	*Good Fit*
_sb_ χ²	150.73	82.87	*-*	*-*
CFI	0.97	0.98	*≥ 0.90*	*Good Fit*
NFI	0.96	0.98	*≥ 0.90*	*Good Fit*
IFI	0.97	0.98	*≥ 0.90*	*Good Fit*
RFI	0.95	0.97	*≥ 0.90*	*Good Fit*
AGFI	0.87	0.92	*≥ 0.80*	*Good Fit*
GFI	0.92	0.96	*≥ 0.90*	*Good Fit*
RMR	0.05	0.04	*≤ 0.08*	*Good Fit*

*RMSEA* Root Mean Square Error of Approximation, *CFI* Comparative Fit Index, *NFI* Normed Fit Index, *IFI* Incremental Fit Index, *RFI* Relative Fit Index, *AGFI* Adjusted Goodness-of-Fit Index, *GFI* Goodness-of-Fit Index, *RMR* Root Mean Square Residual, *BM* Model fit before modification, *AM* Model fit after modification

### Internal consistency reliability

The internal consistency of the Persian version of the Perceived Parental Phubbing Scale (PPPS) was assessed using Cronbach’s alpha, McDonald’s omega, and Guttman’s lambda coefficients. The results indicated good reliability across all three indices: Cronbach’s *α* = 0.90, McDonald’s *ω* = 0.901, and Guttman’s *λ* = 0.853. These values exceed the commonly accepted thresholds for internal consistency (≥ 0.80), demonstrating the good reliability of the scale in the present sample.

### Convergent validity

The Perceived Parental Phubbing Scale (PPPS) demonstrated good convergent validity, as indicated by significant positive correlations with smartphone addiction (*r* = .29, *p* < .01), loneliness (*r* = .27, *p* < .01), and fear of missing out (*r* = .42, *p* < .01), confirming the theoretical relevance of the construct (see Table 3).

**Table 3:** Descriptive statistics and two-tailed correlations among study variables

	M	SD	PPPS	SABAS	ULS-8	FoMo
PPPS	17.13	6.88	1			
SABAS	17.63	6.82	0.29**	1		
ULS-8	16.41	4.31	0.28**	0.28**	1	
FoMo	20.01	7.24	0.42**	0.54**	0.37**	1

*PPPS* Perceived Parental Phubbing Scale, *SABAS* Smartphone Application-Based Addiction Scale, *ULS-8* UCLA Loneliness Scale-Short Form, *FoMo* Fear of Missing Out Scale, *M* Mean, *SD* Standard Deviation

**p < .01

### Group differences

Further analyses revealed that participants with a history of psychological disorders (*n* = 20) reported significantly higher PPPS scores (*M* = 21.10, *SD* = 3.97) than did those without such a history (*n* = 408; *M* = 16.93, *SD* = 6.94), *t* (426) = 6.77, *p* = .008. Additionally, a one-way ANOVA revealed significant differences in PPPS scores across marital status groups, *F* (3, 425) = 3.65, *p* = .013, with participants in a relationship reporting the highest mean score (*M* = 20.03, *SD* = 8.18) and divorced participants reporting the lowest score (*M* = 13.00, *SD* = 2.83), as shown in Table 4.

**Table 4:** Differences in perceived parental phubbing scores across sociodemographic characteristics

	*n*	M	SD	Test statistic
Gender Status				*t(*426*)* = 0.04, *p* = *.*966
Female	303	17.14	6.96	
Male	125	17.10	16.71	
Psychological disorder				*t(*426*)* = 6.77, *p* = *.*008
No	408	16.93	6.94	
Yes	20	21.10	3.97	
Psychological disorder in the family				*t(*426*)* = 1.64, *p* = *.*101
No	402	16.99	6.81	
Yes	26	19.27	7.82	
Residence				*t(*426*)* = 0.74, *p* = *.*460
Rural	47	17.83	7.72	
Urban	381	17.04	6.78	
Marital Status				*F(*3, 425*)* = 3.65, *p* = *.*013
Single	189	16.31	6.84	
Married	203	17.55	6.63	
In relationship	31	20.03	8.18	
Divorced	5	13.00	2.83	
Educational Status				*F(*3, 425*)* = 0.25, *p* = *.*860
Associate degree	115	16.81	7.18	
Bachelor	227	17.39	6.79	
Master	81	16.88	6.76	
PhD	5	16.40	7.70	

## Discussion

The current study aimed to translate, adapt, and validate the Persian version of the Perceived Parental Phubbing Scale (PPPS) among Iranian university students, providing a valid and reliable tool for both empirical research and practical psychological applications.

Firstly, confirmatory Factor Analysis (CFA) supported a unidimensional factor structure for the Persian PPPS. This structure is consistent with the original Partner Phubbing Scale [13] and with the validation conducted by Li et al. [84] among Chinese parents. Unlike these earlier studies, which primarily relied on parental self-reports, the present research examined young adults’ perceptions of parental phubbing. The single-factor model demonstrated an acceptable fit across all indices, reinforcing the conceptual coherence of the construct and supporting the appropriateness of the unidimensional framework.

After confirming the factor structure, we evaluated internal consistency and examined correlations with theoretically related constructs. The Persian PPPS demonstrated strong reliability, comparable to the original scale and in line with prior studies [15, 22, 23, 85–88]. As anticipated, PPPS scores were positively associated with smartphone addiction, loneliness, and FoMO. These associations align with previous empirical findings and provide additional evidence for convergent validity.

As part of a comprehensive psychometric evaluation, it is also important to explore how scores vary across different groups. To do so, we examined potential differences across sociodemographic and psychological characteristics. Gender differences were not significant, suggesting that male and female students perceive parental phubbing in similar ways [15]. This result differs from studies conducted with adolescents [2, 30, 63, 89, 90]. One possible explanation for these discrepancies lies in the developmental focus of prior research. While many earlier studies targeted children and adolescents, the current research centers on young adults. Given these developmental differences, future research should further explore how age and life stage moderate perceptions of parental phubbing.

Psychological health was another important factor. Participants who reported psychological disorders had higher PPPS scores, suggesting greater sensitivity to parental inattention. This observation is consistent with previous research suggesting a vicious cycle in which psychiatric symptoms increase sensitivity to perceived social rejection and heighten the likelihood of interpreting interpersonal cues, including parental phubbing, as signals of exclusion or devaluation [91–96]. These dynamics may, in turn, intensify psychological disorders over time [97]. Taken together, these patterns suggest that perceived parental phubbing is not merely a behavioral observation but also a social-cognitive experience shaped by individual psychological predispositions. Future research could further investigate how specific psychological symptoms, such as anxiety or depressive tendencies, shape individuals’ interpretations of parental smartphone use.

Relational context also emerged as a relevant factor. Participants who reported being in a romantic relationship exhibited the highest PPPS scores, whereas divorced participants reported the lowest. Married and single individuals reported comparable intermediate levels. While the small number of divorced participants limits generalizability, the overall pattern may reflect the heightened salience of parental interactions among individuals currently navigating intimate partnerships. One possible explanation is that individuals in active relationships may be more attuned to relational norms and thus more sensitive to perceived neglect or inattention from parental figures. Conversely, divorced individuals may experience reduced parental engagement or interpret parental behavior with lower emotional reactivity, though this interpretation remains speculative.

Other demographic variables, including residential status (urban versus rural), educational level, and the presence of psychological disorders among immediate family members, did not show significant differences. These null findings suggest that perceived parental phubbing is more closely linked to individual-level psychopathological and relational processes than to broad demographic factors. Notably, the observed group differences may reflect the potential role of primary vulnerabilities and attachment-based expectations as mechanisms shaping the perception of being phubbed.

### Limitations

Although the current study provides valuable insights into the assessment of perceived parental phubbing and introduces a validated and reliable tool tailored for Iranian university students, several limitations should be acknowledged.

First, the cross-sectional design limits the ability to establish causal relationships or longitudinal effects between PPPS and its psychological correlates (e.g., loneliness, FoMO, smartphone addiction) or demographic variations. Future longitudinal studies are needed to clarify the temporal dynamics and directionality of these associations.

Second, the sample consisted exclusively of Iranian university students aged over 18, which may restrict the generalizability of the findings to other populations. Cross-cultural replications with more representative samples are recommended to validate the scale’s applicability.

Third, the reliance on convenience sampling and self-report measures introduces potential biases, such as social desirability or recall bias. Since participants’ subjective perceptions may not fully align with actual parental phubbing behaviors, future studies could benefit from multimethod assessments (e.g., observational data or parent-reported measures).

Fourth, the study’s group comparisons should be interpreted with caution due to unequal sample sizes across categories. This imbalance may reduce the statistical power to detect true differences and limit the generalizability of findings for smaller subgroups.

Finally, although the scale demonstrated good internal consistency, the exclusion of reverse-coded items, originally intended to improve clarity, may reduce the ability to control for acquiescence bias. Future versions of the scale could explore alternative approaches to address this concern while preserving psychometric quality.

## Conclusion

This study validated and introduced the Persian version of the Perceived Parental Phubbing Scale (PPPS) among Iranian university students, providing a culturally adapted tool for measuring perceived neglect in parent–child relationships. The findings highlight parental phubbing as a significant psychosocial factor in the Iranian context, with considerable implications for students’ emotional well-being. Unlike prior studies, predominantly conducted in East Asia, our results revealed no gender differences in perceived parental phubbing, suggesting potential cultural or developmental divergences in interpreting these behaviors. The study also identified specific subgroups, particularly individuals with psychological disorders and those in romantic relationships, as being more sensitive to parental phubbing, highlighting the role of individual vulnerability and relational dynamics. By enabling cross-cultural comparisons and identifying at-risk groups, the validated PPPS establishes a foundation for forthcoming inquiries into digital parenting practices and their psychological impact. These insights may contribute to the development of responsive interventions aimed at promoting mindful smartphone use within families.

## Supplementary Information

Supplementary Material 1.

Supplementary Material 2.

## Data Availability

The datasets used and analysed during the current study are available from the corresponding author upon reasonable request.
