# Supplementary material for: Psychometric evaluation of the Perceived Parental Phubbing Scale (PPPS) among Iranian university students: associations with psychosocial factors and group differences
Source: BMC Public Health. 2026 Feb 4;26:810. doi: 10.1186/s12889-026-26498-y (PMC12964594; doi:10.1186/s12889-026-26498-y)
Supplement: Supplementary file 1 — Supplementary Material 1. [file 12889_2026_26498_MOESM1_ESM.pdf]

Perceived Parental Phubbing Scale (PPPS)

English Version of the Perceived Parental Phubbing Scale (PPPS)

Please indicate how often each statement applies to your experience with your parents, using the following 5-point Likert scale: **1 = Never** **2 = Rarely** **3 = Sometimes** **4 = Often** **5 = Always**

1. During meals, when I am with my parents, they pull out and check their phones
2. My parents place their phones somewhere visible when they are with me.
3. When I am with my parents, they hold their phones in their hands.
4. When their phones ring or beep, my parents check them even if we are in the middle of a conversation.
5. My parents glance at their phones while talking to me.
6. During leisure time, which I could spend with both my mother and father, they use their phones.
7. When I talk to my parents, they are using their phones at the same time.
8. When we go out together, my parents use their phones.
9. If there is a lull in our conversation, my parents check their phones.

مقیاس ادراک شده‌ی فابینگ والدین (PPPS)

لطفاً مشخص کنید هر یک از عبارات زیر تا چه اندازه در مورد تجربه‌ی شما با والدین‌تان صدق می‌کند. برای پاسخ، از مقیاس پنج‌درجه‌ای زیر استفاده کنید:

۱ =هیچ‌وقت **۲ = به ندرت** **۳ = بعضی مواقع** **۴ = بیشتر اوقات** **۵ = همیشه**

۱. در زمان صرف وعده‌های غذایی که با والدینم هستم، آن‌ها گوشی خود را بیرون آورده و آن را چک می‌کنند.
۲. والدینم گوشی خود را در جایی قرار می‌دهند که هنگام با من بودن آن را ببینند.
۳. وقتی با والدینم هستم، آن‌ها گوشی خود را در دست نگه می‌دارند.
۴. وقتی گوشی والدینم زنگ می‌خورد یا صدایی می‌دهد، حتی اگر در میانه‌ی گفتگو باشیم، آن را چک می‌کنند.
۵. والدینم هنگام صحبت با من نگاهی به گوشی خود می‌اندازند.
۶. در اوقات فراغتی که می‌توانیم با هم بگذرانیم، آن‌ها از تلفن همراه خود استفاده می‌کنند.
۷. وقتی با پدر و مادرم صحبت می‌کنم، آن‌ها همزمان از تلفن همراه خود استفاده می‌کنند.
۸. وقتی با هم بیرون می‌رویم، آن‌ها از تلفن همراه خود استفاده می‌کنند.
۹. اگر حین صحبت سکوتی پیش بیاید، والدینم گوشی خود را چک می‌کنند.
