# Supplementary material for: Psychometric evaluation of the Perceived Parental Phubbing Scale (PPPS) among Iranian university students: associations with psychosocial factors and group differences
Source: BMC Public Health. 2026 Feb 4;26:810. doi: 10.1186/s12889-026-26498-y (PMC12964594; doi:10.1186/s12889-026-26498-y)
Supplement: Supplementary file 2 — Supplementary Material 2. [file 12889_2026_26498_MOESM2_ESM.pdf]

Demographic Questions

Thank you for participating in this study. The purpose of this questionnaire is to collect general demographic information that will help us better understand the characteristics of the participants. Your responses will be kept strictly confidential and will be used for research purposes only. Please answer the following questions by selecting the option that best describes you.

1. **Please indicate your gender:**
    1. Male
    2. Female
2. **Do you have a history of psychological disorders?**
    1. No
    2. Yes
3. **Does your family have a history of psychological disorders?**
    1. No
    2. Yes
4. **Which type of area do you currently live in?**
    1. Rural
    2. Urban
5. **Please indicate your marital status:**
    1. Single
    2. Married
    3. In a relationship
    4. Divorced
6. **What is your current level of education?**
    1. Associate degree
    2. Bachelor
    3. Master
    4. Phd
